# Group I Metabotropic Glutamate Receptors and Interacting Partners: An Update

**DOI:** 10.3390/ijms23020840

**Published:** 2022-01-13

**Authors:** Li-Min Mao, Alaya Bodepudi, Xiang-Ping Chu, John Q. Wang

**Affiliations:** 1 Department of Biomedical Sciences, School of Medicine, University of Missouri-Kansas City, Kansas City, MO 64108, USA; maol@umkc.edu (L.-M.M.); abc47@mail.umkc.edu (A.B.); chux@umkc.edu (X.-P.C.); 2 Department of Anesthesiology, School of Medicine, University of Missouri-Kansas City, Kansas City, MO 64108, USA

**Keywords:** interaction, mGlu, glutamate, phosphorylation, ERK, Fyn, Homer

## Abstract

Group I metabotropic glutamate (mGlu) receptors (mGlu1/5 subtypes) are G protein-coupled receptors and are broadly expressed in the mammalian brain. These receptors play key roles in the modulation of normal glutamatergic transmission and synaptic plasticity, and abnormal mGlu1/5 signaling is linked to the pathogenesis and symptomatology of various mental and neurological disorders. Group I mGlu receptors are noticeably regulated via a mechanism involving dynamic protein–protein interactions. Several synaptic protein kinases were recently found to directly bind to the intracellular domains of mGlu1/5 receptors and phosphorylate the receptors at distinct amino acid residues. A variety of scaffolding and adaptor proteins also interact with mGlu1/5. Constitutive or activity-dependent interactions between mGlu1/5 and their interacting partners modulate trafficking, anchoring, and expression of the receptors. The mGlu1/5-associated proteins also finetune the efficacy of mGlu1/5 postreceptor signaling and mGlu1/5-mediated synaptic plasticity. This review analyzes the data from recent studies and provides an update on the biochemical and physiological properties of a set of proteins or molecules that interact with and thus regulate mGlu1/5 receptors.

## 1. Introduction

l-Glutamate (glutamate) is a key neurotransmitter in the mammalian brain and is involved in the regulation of various neuronal and synaptic activities through activating two classes of receptors: ionotropic glutamate (iGlu) receptors and metabotropic glutamate (mGlu) receptors. iGlu receptors are ligand-gated ion channels and mediate fast excitatory synaptic transmission. Based on their ligand binding properties (pharmacology) and sequence similarity, iGlu receptors are divided into four subtypes: *α*-amino-3-hydroxy-5-methylisoxazole-4-propionic acid (AMPA) receptors, kainate receptors, *N*-methyl-d-aspartate (NMDA) receptors, and delta receptors [1]. mGlu receptors represent a family of G protein-coupled receptors (GPCRs). A total of eight subtypes (mGlu1–8) are further classified into three functional groups based on their sequence homology, postreceptor signaling connections, and pharmacology [2]. Group I mGlu receptors (mGlu1 and mGlu5 subtypes) are coupled to heterotrimeric Gα_q/11_ proteins. Ligand stimulation of these subtypes leads to activation of phospholipase Cβ (PLCβ) and a subsequent increase in hydrolysis of membrane-bound phosphoinositide (PI). Increased PI hydrolysis yields diacylglycerol (DAG), which activates protein kinase C (PKC), and inositol-1,4,5-trisphosphate (IP_3_), which triggers an intracellular release of Ca^2+^. Active PKC and released Ca^2+^ can then modulate diverse downstream signaling molecules and pathways to finetune synaptic transmission and plasticity. Group II (mGlu2/3) and III (mGlu4/6–8) receptors are coupled to Gα_i/o_ proteins. They thus inhibit adenylyl cyclase, which reduces cAMP formation and protein kinase A activity [2]. Of note, group I mGlu receptors are often postsynaptic and are concentrated in the postsynaptic density (PSD) (see below), as opposed to group II/III receptors, which are either enriched both presynaptically and postsynaptically or localized predominantly at presynaptic sites. As such, group I receptors modulate excitatory synaptic transmission and plasticity preferentially via a postsynaptic mechanism.

Group I mGlu receptors exhibit a typical membrane topology for a GPCR. Namely, they contain an extracellular *N*-terminus, an intracellular *C*-terminus (CT), three intracellular loops, and seven transmembrane domains. Intracellular domains provide the receptors with a spacious structural basis for direct protein–protein interactions. Several categories of membrane-bound or cytoplasmic proteins access and bind to group I receptors [3,4]. Among these binding proteins are protein kinases, such as mitogen-activated protein kinases (MAPKs), Src family kinases (SFKs), Ca^2+^/calmodulin-dependent protein kinase II (CaMKII), and PKC [3,4,5,6]. These kinases are present within the PSD microdomain, bind to intracellular domains of mGlu1/5 receptors, especially the CT region, and phosphorylate specific residues on the receptors. Several scaffolding or adaptor proteins involved in endocytosis and/or exocytosis of membrane-bound receptors also interact with mGlu1/5. As expected, dynamic changes in protein–protein interactions have functional consequences. By either constitutive or activity-dependent binding to mGlu1/5, binding partners modulate the clustering, trafficking (internalization and externalization), anchoring, and turnover of the receptors. As such, protein–protein interactions control the efficacy of mGlu1/5 coupling to various postreceptor signaling pathways and mGlu1/5-mediated synaptic plasticity. Recent studies have made significant progress on the biochemistry and physiology of protein–protein interactions between mGlu1/5 receptors and their interacting partners. This review summarizes the data primarily from the last five years of research and attempts to provide an update on this important topic. 

## 2. Group I mGlu Receptors

Rodent *Grm1* and human *GRM1* genes encode mGlu1 receptors [7]. Alternative splicing of these genes produces several mGlu1 splice variants, including mGlu1a (mGlu1α), mGlu1b (mGlu1β1), mGlu1f (mGlu1β2), mGlu1d (mGlu1γ), and mGlu1E55 (mGlu1δ) [7]. mGlu1a is a long-form variant, and full-length rat mGlu1a has 1199 amino acids (accession numbers: NP_058707/NCBI and P23385/UniProtKB/Swiss-Prot) [8,9]. Similarly, genes encoding mGlu5 receptors are *Grm5* (rat and mouse) and *GRM5* (human). Like mGlu1, mGlu5 has splice variants: mGlu5a, mGlu5b, and mGlu5d. mGlu5a is a long-form variant with 1171 amino acids in rat mGlu5a (NP_058708/NCBI) [10]. As class C GPCRs, mGlu1/5 receptors have seven transmembrane domains, which give rise to an extracellular N-terminus, three intracellular loops, and a CT tail protruding intracellularly. A large CT domain in the long isoforms of group I mGlu (mGlu1a/5a/5b) is noteworthy. In fact, the rat mGlu1a CT contains a total of 359 amino acids (K841-L1199), while a short-form variant mGlu1b has only 68 CT residues. The rat mGlu5a CT is comprised of 345 residues (K827-L1171), and mGlu5b contains an additional 32-residue fragment inserted into the CT region [10]. The mGlu1/5 CT is important for modulating G protein coupling. Additionally, the mGlu1/5 CT region is readily accessible to submembranous binding partners and represents a major intracellular domain for functional protein–protein interactions [3,4].

In addition to the canonical Gα_q/11_-mediated PLCβ-PI pathway, mGlu1/5 receptors modulate a range of other effectors/cascades via either G protein (Gα_q_, Gα_i/o_, or Gα_s_)-dependent or -independent mechanisms. Depending on the cell type or specific neuronal population surveyed, mGlu1/5 receptors induce positive responses from cAMP, arachidonic acid, phospholipase D, multiple protein kinases including MAPKs, tyrosine phosphatase, and L-type voltage-sensitive Ca^2+^ channels (reviewed in [2,9,11]). mGlu1/5 receptors also inhibit a variety of K^+^ channels [9]. Regarding synaptic plasticity, group I receptors are required for inducing long-lasting forms of synaptic plasticity, long-term depression (LTD), and long-term potentiation (LTP), at multiple glutamatergic synapses in order to exert the long-lasting regulation of neuronal excitability [11]. The distribution pattern of the mGlu1 receptor in the brain is characterized by extensive expression in Purkinje cells of the cerebellum, while other brain regions also express the receptor at variable levels [9]. The mGlu5 receptor is abundant in many brain regions, including the hippocampus, striatum, and cerebral cortex. In addition to neurons, mGlu1/5 receptors are expressed in glial cells (astrocytes, oligodendrocytes, and microglia) [12], neural stem/progenitor cells [13], and peripheral organs [9,14]. Of note, at the subcellular/subsynaptic level, mGlu1/5 receptors are primarily postsynaptic at excitatory synapses and are seemingly concentrated in perisynaptic and extrasynaptic areas. As such, mGlu1/5 may preferentially respond to glutamate that spills over from the synaptic cleft due to prolonged or repetitive stimulation [7].

## 3. MAPKs

The MAPK family includes three subclasses: extracellular signal-regulated kinases (ERKs), c-Jun *N*-terminal kinases/stress-activated protein kinases (JNK/SAPKs), and p38 MAPKs [15]. As serine/threonine protein kinases, MAPKs are engaged in phosphorylating specific serine or threonine residue(s) on substrates, leading to dynamic regulation of the expression and function of phosphorylation-modified substrates. All MAPKs share some basic biochemical properties of phosphorylation, e.g., binding to a similar domain and phosphorylating a consensus proline-directed motif (S/TP), although the primary amino acid sequence preferred around binding and phosphorylation sites can be heterogeneous [16]. Noticeably, in addition to the cytoplasmic and nuclear distribution, ERKs and JNKs are found in neuronal peripheral structures, including dendritic spines and synapses, and ERK is present in the defined PSD region [17]. This PSD presence indicates a likely existence of some substrates of MAPKs at synaptic sites, in addition to those well-known substrates in the nucleus. 

Increasing evidence supports synaptic mGlu1/5 receptors to be sufficient substrates of ERK. Yang et al. [18] found that mGlu1a is among local substrates of the synaptic pool of ERK2. In protein–protein binding assays in vitro with purified proteins, ERK2 in either an inactive or active form directly bound to a CT domain (the first 45 amino acids in the membrane proximal region, K841-N885) of recombinant mGlu1a. In the rat cerebellum where mGlu1 is enriched while mGlu5 is almost lacking [19,20], native ERK2 and mGlu1a proteins interacted with each other at synaptic sites in vivo as detected by co-immunoprecipitation with synaptosomal samples. Active ERK2 showed the ability to constitutively phosphorylate mGlu1a CT at a cluster of serine residues, including S1147, S1154, and S1169. Notably, this basal phosphorylation has functional sequences as it is critical for 1) maintaining adequate surface expression of mGlu1a receptors, 2) triggering the canonical mGlu1a-IP_3_ signaling pathway, and 3) linking mGlu1a to Src. In addition to mGlu1a, the mGlu5 receptor is subject to regulation by ERK. A previous study found that constitutively active MEK (MAPK kinase), which activates ERK, increased mGlu5 CT phosphorylation at S1126 in HEK293T cells as detected by a phospho- and site-specific antibody [21]. In these transfected HEK293T cells, i.e., a cell line with expression of recombinant ERK1 and mGlu5 proteins through transfection, ERK1 and mGlu5 were co-immunoprecipitated. In cultured mouse cortical neurons, an MEK inhibitor reduced basal S1126 phosphorylation of native mGlu5. Given that S1126 resides within the highly conserved Homer binding motif on mGlu5 (rat: 1123TPPSPF), phosphorylation of S1126 as well as T1123 exerts a significant impact on the Homer-mGlu1/5 binding. Consistently, recombinant ERK1 was recently confirmed to directly bind to mGlu5 CT in vitro, and endogenous ERK1 and mGlu5 formed complexes in rat striatal neurons enriched with mGlu5 receptors [22]. Under basal conditions, the ERK1-mediated mGlu5 phosphorylation appears to be critical for mGlu5 signaling to IP_3_ production [22]. 

Emerging evidence links the ERK-directed mGlu5 phosphorylation to drugs of abuse. Binge-alcohol intake increased ERK phosphorylation in the bed nucleus of the stria terminalis (BNST) of mice [23]. Point mutations of mGlu5 that caused the lack of phosphorylation at T1123/S1126 by proline-directed kinases including ERK [21,24] led to excessive alcohol drinking [23]. These transgenic mice were also insensitive to alcohol aversion, which was mimicked by local inhibition of ERK within the BNST. Thus, the ERK-mediated mGlu5 phosphorylation in the BNST seems to curb excessive alcohol consumption. The ERK-dependent mGlu5 phosphorylation also suppresses the rewarding effect of methamphetamine as transgenic global disruption of mGlu5 T1123/S1126 phosphorylation augmented the methamphetamine-induced conditioned place preference [25]. Of note, T1123/S1126 phosphorylation is not involved in the methamphetamine-induced acute locomotion or locomotor sensitization but is necessary for the reinforcing property of methamphetamine as tested under operant-conditioning procedures [25] and for cocaine sensitization [24]. In addition, repeated nicotine injections increased the interaction of active ERK with mGlu5 in the rat nucleus accumbens (NAc) [26]. An interfering peptide that specifically disrupts the ERK-mGlu5 binding reduced the repeated nicotine-induced increase in locomotor activity, indicating a role of the ERK–mGlu5 interaction in processing the nicotine effect [26]. In sum, the ERK–mGlu5 interplay plays pivotal and different roles in drug addiction, depending on drug types, brain regions, and administration paradigms. 

## 4. Fyn

In addition to serine and threonine, tyrosine is a site subjected to phosphorylation. In general, tyrosine phosphorylation is catalyzed by tyrosine kinases, including both receptor and nonreceptor tyrosine kinases. By phosphorylating tyrosine residue(s), tyrosine kinases modulate the expression and function of proteins. A subfamily of nonreceptor tyrosine kinases that has been mostly studied in the regulation of glutamate receptors is the SFK [27]. Of nine SFK members, five are expressed in the brain [27]. Among these five members (Fyn, Src, Yes, Lyn, and Lck), Fyn (isoform 1, also known as FynB) and Src are of particular interest. Both are particularly enriched at synaptic sites and are therefore considered to target local substrates for the sake of modulating synaptic transmission and plasticity [28]. In fact, a growing body of evidence supports the roles of Fyn and Src in phosphorylating iGlu receptors and modulating their biochemistry, biophysics, and physiology [27,29]. Emerging evidence also identifies Fyn as a key SFK member for the regulation of group I mGlu receptors.

**Figure 1:**
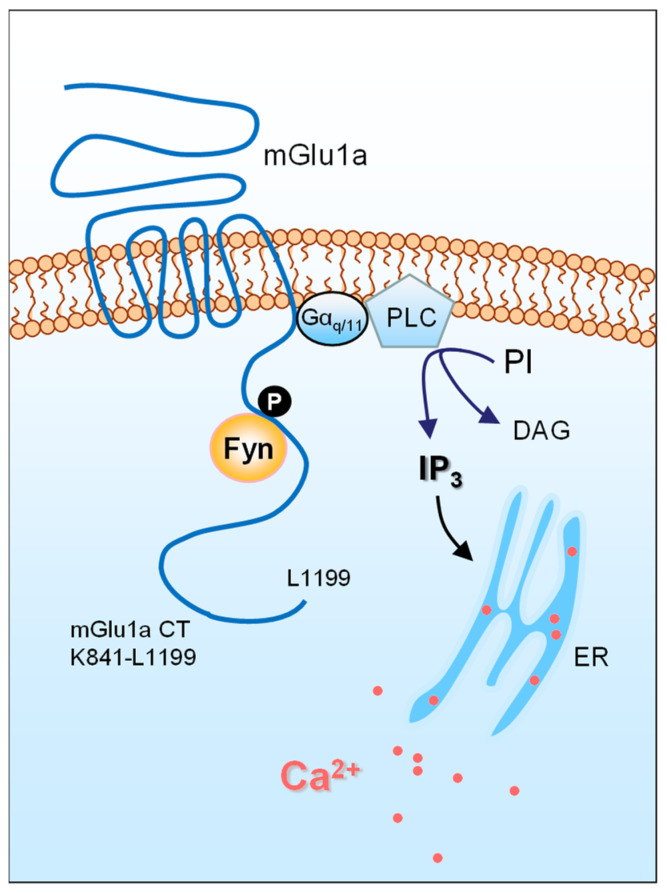
A schematic diagram illustrating an interaction between Fyn and a metabotropic glutamate receptor. The mGlu1a receptor is coupled to Gα_q/11_ proteins. Ligand stimulation of mGlu1a receptors activates PLC and subsequently hydrolyzes PI to yield DAG and IP_3_, which triggers an intracellular release of Ca^2+^ from the endoplasmic reticulum (ER). Released Ca^2+^ could modulate various downstream signaling molecules and pathways to control synaptic transmission and plasticity. The SFK member Fyn binds to the mGlu1a CT. Through phosphorylating a tyrosine residue in the mGlu1a CT, Fyn supports surface trafficking and IP_3_ signaling of the receptor.

mGlu1a was recently targeted for possible tyrosine phosphorylation by Fyn [30]. It is noted that the CT domain of mGlu1a is the only region, among all intracellular domains, where tyrosine residues are present. In the presence of active Fyn, a conserved tyrosine site in the mGlu1a CT was sufficiently phosphorylated in vitro, implying that mGlu1a is likely a new substrate of Fyn (Figure 1) [30]. As seen in many known Fyn substrates, purified Fyn directly bound to mGlu1a CT in binding assays in vitro. Similarly, native Fyn and mGlu1a formed complexes in the rat cerebellum, where they are both abundantly expressed and are enriched in the PSD. The Fyn–mGlu1a interaction is functionally relevant as constitutively active Fyn, through its binding to mGlu1a, drives surface expression and IP_3_ signaling of mGlu1a in cerebellar neurons and HEK293T cells [30]. Taken together, a previously unrecognized Fyn–mGlu1a coupling seems to exist in cerebellar neurons, and Fyn is among the mGlu1a-interacting partners that profoundly regulate the receptor.

mGlu5 receptors were tyrosine-phosphorylated in the rat striatum [31] and the mouse forebrain [32]. A series of pharmacological studies conducted recently in vivo reveals that tyrosine phosphorylation of mGlu5 may involve Fyn. Blocking Gα_i/o_-coupled dopamine D_2_ receptors with a D_2_ blocker eticlopride induced autophosphorylation (activation) of Fyn but not Src in the adult rat striatum, indicating an inhibitory linkage from D_2_ receptors to Fyn in the region [33]. Interestingly, eticlopride simultaneously elevated tyrosine phosphorylation of mGlu5 and enhanced the trafficking of mGlu5 into the PSD, which was blocked by an SFK inhibitor. Thus, Fyn appears to mediate tyrosine phosphorylation and synaptic delivery of mGlu5 receptors in striatal neurons, which is under the inhibitory modulation by D_2_ receptors under normal conditions. In another study, Fyn and Src were found to interact with mGlu5 in rat striatal neurons in co-immunoprecipitation assays [34]. Prolonged social isolation, an animal paradigm modeling depression, selectively increased the Fyn–mGlu5 interaction. Social isolation also enhanced surface expression of striatal mGlu5 receptors, which was reduced by an SFK inhibitor. Thus, Fyn and mGlu5 interact with each other in neurons, which is sensitive to a chronic stressor (social isolation) and may play a role in the development of depression [34]. Nevertheless, future studies will need to investigate whether Fyn directly interacts with mGlu5 and will map accurate tyrosine site(s) among only a few tyrosine residues in the mGlu5a CT that accept phosphorylation by Fyn. 

## 5. CaMKII and PKC

CaMKII is a synapse-enriched serine/threonine kinase. Early pharmacological studies identified CaMKII as a required kinase for the internalization and homologous or heterologous desensitization of mGlu1a receptors [35,36]. In in vitro binding assays with purified recombinant proteins, CaMKIIα directly bound to mGlu1a CT [37]. Through phosphorylating mGlu1a CT T871, CaMKIIα triggers a negative feedback loop controlling the agonist-induced desensitization of the receptor. Similarly, inactive CaMKIIα directly bound to the membrane proximal region of mGlu5 CT in vitro, which was competitively inhibited by Ca^2+^/calmodulin, and endogenous CaMKIIα and mGlu5 were associated with each other as they were present in rat striatal co-immunoprecipitated complexes [38]. Marks and co-workers [39] confirmed that native mGlu5 is a bona fide component of the CaMKIIα complexes immunoprecipitated from the mouse forebrain. Furthermore, they observed that activated and T286-autophosphorylated CaMKIIα bound to mGlu5a CT in vitro, which was disrupted by Ca^2+^/calmodulin. In HEK293A cells, binding of active CaMKIIα to mGlu5 CT increased basal mGlu5a surface expression and altered the mGlul/5-stimulated Ca^2+^ mobilization pattern, i.e., reducing and prolonging the initial peak and duration of the mGlu5-mediated Ca^2+^ rise, respectively [39]. In addition to the CT region, CaMKIIα appears to interact with the second intracellular loop (IL2) of both mGlu1a and mGlu5a. In a proteomic screen, CaMKIIα was identified in a pool of proteins co-precipitated with a cell-permeable mGlu1/5 IL2 peptide in cultured mouse cortical and striatal neurons [40]. CaMKIIα overexpression positively regulated the agonist-stimulated mGlu1a/5a endocytosis but selectively attenuated mGlu5a—although not mGlu1a—stimulated ERK1/2 phosphorylation in transfected HEK293 cells. 

PKC is a kinase that has been most extensively studied in the early studies for its roles in phosphorylating mGlu1/5 and thereby modulating Ca^2+^ transient patterns, internalization, and homologous and heterologous desensitization of the receptors following ligand stimulation (reviewed in [41]). New studies have extended the existence and physiology of the PKC–mGlu1/5 interaction in different systems. A PKCε inhibitor increased mGlu5 surface expression in the rat NAc under basal conditions [42]. Activation of PKCε mediated the agonist-independent (no mGlu5 agonist present) and agonist-dependent internalization of mGlu5 in acute rat NAc slices. Thus, this particular PKCε isoform regulates the trafficking and subcellular distribution of mGlu5 in the NAc under both basal and stimulated conditions. In addition, in cultured rat astrocytes, knockdown of PKCε expression with an shRNA approach altered Ca^2+^ oscillatory responses to mGlu5 activation, indicating that intact PKCε is required for generating normal Ca^2+^ oscillations in response to mGluR5 stimulation in astrocytes [43]. 

## 6. Scaffolding Proteins

Homer is a synaptic scaffolding protein and a known binding partner of group I mGlu receptors [44]. By binding to a proline-rich motif on mGlu1a CT (rat: 1151TPPSPF) and mGlu5 CT (rat: 1123TPPSPF) through Homer’s *N*-terminal EVH1 domains, the Homer family of proteins controls organization, localization, and function of the receptors [45,46]. Functional roles of Homers in regulating mGlu1/5 signaling were further profiled recently. In astrocytes, the long form of crosslinking Homers (Homer1b/c) was clustered with mGlu5 receptors within the areas showing intense Ca^2+^ activity [47]. By interacting with mGlu1/5, Homer1b amplified the mGlu1/5 agonist DHPG-induced intracellular Ca^2+^ signaling and glutamate release from astrocytes, while a dominant-negative short variant, Homer1a, which is activity-dependently induced [46], seemed to play an opposite role. In mouse primary visual cortical neurons, Homer1a induction and Homer1a–mGlu5 interactions were critical for the experience-dependent weakening of excitatory synapses, an input-specific metaplasticity that maintains stability in sensory neural networks [48]. The Homer1a–mGlu5 interaction also plays a novel role in homeostatic scaling-down of excitatory synapses, a global form of synaptic plasticity, during sleep [49] and in strengthening ocular dominance in the visual cortex [50]. In addition, phosphorylation of mGlu5 receptors at the Homer-binding motif (T1123 and S1126) enhanced Homer–mGlu5 crosslinking in the mouse spinal cord, contributing to the complete Freund’s adjuvant-induced inflammatory pain [51]. 

The Homer–mGlu1/5 binding is a regulated event. Recently, active CaMKIIα was found to phosphorylate long Homers (Homer1/2) at specific serine sites, which subsequently reduced interactions of Homers with mGlu5 receptors [52]. Since disrupted Homer-mGlu5 scaffolds contribute to Fragile X Syndrome, elevated CaMKIIα activity and resultant hyperphosphorylation of Homers may serve as a molecular mechanism underlying disrupted Homer–mGlu5 interactions and relevant phenotypes of the disease [52,53]. Hu et al. [54] showed that brain-derived neurotrophic factor (BDNF) regulates Homer binding to group I receptors. In rat sympathetic neurons from the superior cervical ganglia, BDNF activation of the tropomyosin-related kinase receptor B recruited a downstream proline-directed kinase, ERK, to phosphorylate T1151 and S1154 in the Homer ligand (1151TPPSPF) of heterologously expressed mGlu1 receptors. The outcome was an increase in Homer binding to mGlu1, which ultimately uncoupled the receptor from voltage-gated Ca^2+^ channels.

**Figure 2:**
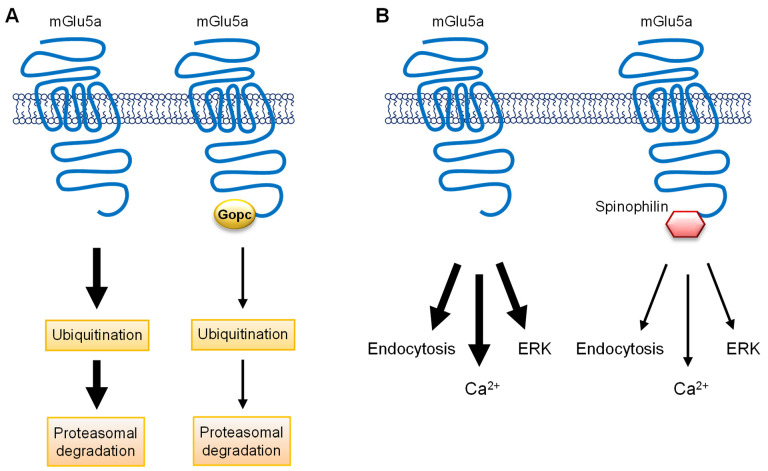
Schematic diagrams illustrating the roles of Gopc and spinophilin in the regulation of mGlu5a receptors. (**A**) The role of Gopc in the regulation of mGlu5a receptors. The mGlu5a receptor is subject to ubiquitination, followed by proteasomal degradation. Gopc via its PDZ domain binds to the PDZ motif on the distal end of mGlu5a CT. This Gopc–mGlu5a interaction inhibits the ubiquitination process of mGlu5a and thereby upregulates the expression level of mGlu5a receptors. (**B**) The role of spinophilin in the regulation of mGlu5a receptors. Spinophilin interacts with the PDZ motif on mGlu5a CT and inhibits the ligand-induced endocytosis of the receptors in cortical neurons. Moreover, spinophilin limits the mGlu5a-mediated signaling events (Ca^2+^ and ERK) in response to agonist stimulation.

PDZ [PSD95/*Drosophila* disc large tumor suppressor (Dlg1)/zonula occludens-1 (zo-1)] domain-containing proteins are another group of synaptic scaffolding proteins that directly bind to a highly conserved PDZ-binding motif at the distal CT end of group I mGlu receptors (1197STL for rat mGlu1 CT; 1169SSL for rat mGlu5 CT) [55]. Recently, additional evidence was obtained regarding the functional interplay between PDZ proteins and mGlu1/5 receptors. Acute knockdown of endogenous Tamalin, a PDZ protein that binds to mGlu1/5 CT [55], inhibited the ligand-induced internalization of mGlu1 and 5 receptors in mouse hippocampal neurons [56]. The Tamalin action involved its interaction with the synaptic scaffolding molecule (S-SCAM), another scaffolding protein in the region. Moreover, a PDZ-blocking peptide that competitively inhibits the Tamalin-mGlu1/5 binding prevented the mGlu1/5 agonist DHPG- and the mGlu5 agonist CHPG-facilitated LTD in the hippocampal CA1 region without affecting LTP [57]. Together, Tamalin plays novel roles in the activity-dependent endocytic trafficking of group I receptors and in the LTD form of synaptic plasticity in hippocampal neurons. In addition to Tamalin, another PDZ protein is involved in trafficking and signaling of mGlu1/5 receptors. The Golgi-associated PDZ and coiled-coil motif-containing protein (Gopc) [also known as protein interacting specifically with Tc10, PIST; and as cystic fibrosis transmembrane conductance regulator (CFTR)-associated ligand, CAL] is a scaffolding protein and interacts with the mGlu1/5 PDZ motif through its PDZ domain [58,59]. By binding to mGlu5a, Gopc inhibited the ubiquitination and degradation of mGlu5a, resulting in an increase in the receptor expression [59] (Figure 2A). Conditional knockout of Gopc reduced the targeting of mGlu5 to the PSD in the mouse hippocampus [60]. Gopc deficiency also impaired mGlu1/5-ERK signaling in astroglial C6 cells, and overexpression of Gopc stabilized mGlu5 expression in the striatum in a rotenone-induced rat model of Parkinson’s disease [61]. Thus, Gopc is required for synaptic targeting, expression, and signaling of mGlu5 receptors in an in vivo setting.

Spinophilin is a modular protein and is comprised of a number of protein–protein binding domains, including a PDZ domain [62]. As a scaffolding protein compartmentalized to the dendritic spine heads, spinophilin interacts with a large number of synaptic proteins, including several GPCRs [63]. A recent study by Di Sebastiano et al. [64] adds spinophilin to the list of group I mGlu receptor-interacting proteins. Spinophilin interacted with the CT and IL2 of group I receptors in transfected HEK293 cells as detected by a proteomic screen and co-immunoprecipitation. Functionally, spinophilin negatively regulated the agonist-induced endocytosis of group I receptors in cultured mouse cortical neurons (Figure 2B). Additionally, endogenous spinophilin limited group I mGlu signaling responses (Ca^2+^ and ERK) to agonist stimulation in cultured cortical neurons (Figure 2B) and was required for the mGlu5-stimulated LTD in mouse hippocampal slices. 

Little was known about the transport of mGlu receptors along microtubules until a recent study conducted by Raynaud et al. [65]. These authors found that the synaptosome-associated protein 23 (SNAP23), a homolog SNARE (soluble N-ethylmaleimide-sensitive-factor attachment protein receptor) protein of SNAP25 [66], is involved in microtubule-dependent postsynaptic trafficking of mGlu1 receptors. In detail, SNAP23 and mGlu1 directly interacted with each other through the CT segment of both proteins. As an adaptor, SNAP23 linked the molecular motor Kif5 kinesin to mGlu1 CT to form a Kif5-SNAP23-mGlu1 complex. This complex enables Kif5 to facilitate vesicular transport of the SNAP23-mGlu1 cargo along dendritic microtubules and to promote exocytosis of mGlu1 receptors to the cell surface in a C6 glioma cell line and cultured rat hippocampal neurons [65]. 

## 7. Proteins Involved in mGlu1/5 Trafficking

Numb is a protein that was originally discovered as a cell fate determinant during neural development in *Drosophila* [67]. In mammalian neurons, Numb can act as an adaptor linking cargos (such as transmembrane receptors) to the clathrin-associated endocytic machinery. In mouse cerebellar Purkinje cells, Numb has been shown to regulate the constitutive expression and ligand-induced internalization and recycling of mGlu1 receptors [68]. Numb (p72 but not p65 isoform) promoted the membrane expression of mGlu1a probably by interacting with mGlu1a and suppressing its internalization [69]. Similarly, Numb (p72 isoform) interacted with mGlu5a in transfected HEK293T cells and the mouse hippocampus as detected by co-immunoprecipitation [70,71]. As an interacting partner, Numb promoted the expression of mGlu5 in the PSD and suppressed ligand-operated mGlu5 internalization. Behaviorally, conditional knockout of Numb in cerebellar Purkinje cells led to deficits in motor coordination [69]. CaMKII-cre-directed conditional knockout of Numb in the mouse forebrain caused autistic-like behaviors, i.e., social interaction deficits [70].

The sorting nexin (SNX) family represents a large number of cytoplasmic and membrane-bound proteins that are involved in various aspects of transporting cargos among different membranous compartments [72]. SNX1 is an SNX member that notably bound to mGlu1a/5a CT in vitro [73], indicating that SNX1 may have a potential to regulate trafficking of mGlu1/5 receptors. In fact, this novel role has recently been demonstrated in neurons. Group I mGlu receptors are known to be recycled back to the cell surface following the ligand-induced internalization, a trafficking step critical for resensitizing these receptors [74]. Sharma et al. [75] found that SNX1 is required selectively for the normal recycling of mGlu1 after the ligand-dependent internalization in cultured mouse hippocampal neurons. The SNX1-mediated recycling of mGlu1 involved Hrs (hepatocyte growth factor-regulated tyrosine kinase substrate), a protein implicated in vesicular trafficking and signaling, and enabled efficient resensitization of the recycled receptors.

Arrestins are a small family of multifunctional adaptor proteins that have four mammalian members: two visual (arrestin1 and arrestin4) and two non-visual members (arrestin2 and arrestin3, also known as β-arrestin1 and β-arrestin2, respectively) [76,77]. β-Arrestins are ubiquitously expressed and are functionally recruited to the CT region of GPCRs after ligand stimulation to inhibit further G protein activation and promote internalization of GPCRs through an arrestin-dependent engagement of clathrin-coated pits [77]. In addition to this classic view over the function of β-arrestins in arresting G protein signaling and inducing receptor desensitization, β-arrestins also mediate alternative GPCR signaling, i.e., linking GPCRs to a particular signaling cascade independent of canonical G protein-coupled signaling [77]. In the context of group I mGlu receptors, β-arrestin2 interacted with mGlu1 in the cortex, hippocampus, and cerebellum and with mGlu5 in the cortex and hippocampus of mice as detected by co-immunoprecipitation [78]. Notably, β-arrestin2 but not Gα_q_-dependent PLCβ-Ca^2+^ signaling links mGlu5 to ERK and protein synthesis in the mouse hippocampus [79]. By linking mGlu5 to protein synthesis, β-arrestin2 mediates the protein synthesis-dependent component of mGlu5-LTD. Similarly, β-arrestin2- but not β-arrestin1-mediated signaling is critical for some forms of synaptic plasticity mediated by mGlu1 in CA3 pyramidal neurons and by mGlu5 in CA1 pyramidal neurons of the mouse hippocampus [78]. These observations of G protein-independent and β-arrestin2-dependent signaling in shaping excitatory synaptic plasticity advance our knowledge about the molecular mechanisms underlying the modulation of excitatory transmission. 

## 8. Other Interacting Partners

The cellular prion protein (PrP^C^) is a normal glycoprotein whose misfolding generates an abnormal isoform, i.e., the scrapie prion protein (PrP^SC^), which is linked to Creutzfeldt–Jakob disease in humans [80]. As a normal membrane-bound and cell surface protein, PrP^C^ is poorly understood for its physiological roles [81]. Matsubara and co-workers [82] recently found that PrP^C^ and mGlu1 interacted with each other in the transfected neuroblastoma (Neuro-2a) cell line and in the mouse cerebellum as assayed by co-immunoprecipitation and fluorescence resonance energy transfer, confirming previous reports in which PrP^C^ interacted with both mGlu1 and 5 receptors in heterologous cells and/or in the mouse brain [83,84,85]. Interestingly, knockdown of PrP^C^ impaired the synchronization of Ca^2+^ oscillations in response to mGlu1 stimulation in Neuro-2a cells. This uncovers a physiological role of PrP^C^ in modulating the phasic pattern of mGlu1-Ca^2+^ signaling. 

In addition to the cell surface expression, GPCRs are localized inside the cell [86]. These intracellular GPCRs are present on mitochondria, endoplasmic reticulum membranes, lysosomes, and various nuclear structures. By modulating distinct signaling systems, intracellular GPCRs play many physiological roles [86]. Among intracellular GPCRs is the mGlu5 receptor. A large portion of mGlu5 is associated with intracellular membranes, such as the inner nuclear membrane (INM) [87]. INM mGlu5 receptors are also coupled to Gα_q/11_ and induce a release of luminal Ca^2+^ and activation of multiple nuclear signaling molecules [87]. However, mechanisms for trafficking and retaining mGlu5 in the particular INM locale are less known. Sergin et al. [88] found that a 25-amino acid sequence within the nucleoplasmic CT domain of mGlu5 (852–876) is sufficient for its trafficking to the INM. Once at the INM, mGlu5 appears to be tethered in place via its CT interactions with the DNA/chromatin. The mGlu5 INM localization domain is a very basic region and is positively charged, which situates well to interact with the negatively charged chromatin. By stably anchoring on the chromatin this way, mGlu5 is poised to execute its functions in regulating Ca^2+^ signals and transcriptional and chromosomal activities in situ.

## 9. Concluding Remarks

The current review highlights the recent progress in analyzing mGlu1/5 interacting partners in their biochemical and physiological properties. The long-form group I mGlu variants, i.e., mGlu1a, mGlu5a, and mGlu5b, are characterized by a unique long CT region, which provides a solid basis for protein–protein interactions and the interaction-dependent modulations. A panel of protein kinases are among mGlu1/5 interacting partners that were extensively investigated in recent years in heterologous expression systems and in neurons. A novel interaction was uncovered and characterized between ERK2 and mGlu1a. The synaptic pool of ERK2 directly binds to mGlu1a CT, which enables ERK2 to phosphorylate a cluster of serine residues and thus promote surface expression of mGlu1a and efficacy of mGlu1a signaling. mGlu5 receptors are also subject to the phosphorylation-dependent regulation by ERK1. Recombinant ERK1 binds to mGlu5 CT in vitro and endogenous ERK1 and mGlu5 form complexes in rat striatal neurons. A serine residue in the distal mGlu5 CT region is phosphorylated by ERK1. Inhibition of ERK reduces the efficacy of mGlu5 in triggering IP_3_ production. In addition to ERK, an SFK member Fyn binds to mGlu1a CT and phosphorylates a conserved tyrosine site in the CT region. In rat cerebellar neurons, the Fyn-mediated mGlu1a phosphorylation is a constitutive event and is essential for surface expression and signaling of the receptor. Of note, while recent studies on phosphorylation of mGlu1/5 receptors by protein kinases have made progress, little is known about phosphatase-mediated dephosphorylation of these novel ERK- and Fyn-sensitive sites under normal or stimulated conditions.

A panel of synaptic scaffolding and adaptor proteins are known to bind to mGlu1/5. Knowledge about the biochemistry and physiology of their interactions with the receptors has been advanced in recent years. For instance, Homer scaffolding proteins bind to mGlu1/5 CT and such interactions are important for an input-specific metaplasticity in visual cortical neurons. The Homer1a–mGlu5 interaction also plays a pivotal role in homeostatic scaling-down of excitatory synapses during sleep. The PDZ-containing proteins, such as Tamalin and Gopc, belong to the scaffolding proteins that bind to mGlu1/5. Recently, Tamalin was found to participate in the activity-dependent endocytic trafficking of mGlu1/5 and in synaptic plasticity (LTD) in hippocampal neurons. Gopc targets mGlu5 to the PSD and links mGlu1/5 to ERK in the mouse hippocampus. 

Several proteins have recently been identified as novel interacting partners of mGlu1/5 receptors. Spinophilin is a scaffolding protein localized in the dendritic spine heads. It interacts with the CT and IL2 of group I mGlu receptors. As a result, spinophilin negatively regulates the ligand-induced endocytosis of group I mGlu receptors and is required for mGlu5-LTD. SNAP23 is another new mGlu1 interacting partner. As an adaptor, SNAP23 takes part in the vesicular transport of the mGlu1 cargo along dendritic microtubules, promoting exocytosis of mGlu1 to the cell surface. In addition to the cell surface protein–protein interaction, mGlu5 was found to interact with the nuclear chromatin, which stably anchors the receptor within the nucleus to carry out functions, such as regulating Ca^2+^ signals and modulating transcriptional and chromosomal activities in situ. It is anticipated that additional mGlu1/5 interacting proteins and molecules are likely to be discovered in future investigations. In-depth exploration and characterization of mGlu1/5 interacting partners will provide insight into the molecular mechanisms underlying the regulation of mGlu1/5 receptors pertaining to their trafficking, subcellular distribution, and function. 

## Data Availability

Not applicable.

## Abbreviations

AMPA	*α*-amino-3-hydroxy-5-methylisoxazole-4-propionic acid
BDNF	Brain-derived neurotrophic factor
BNST	bed nucleus of the stria terminalis
CaMKII	Ca^2+^/calmodulin-dependent protein kinase II
CT	*C*-terminus
DAG	diacylglycerol
ERK	extracellular signal-regulated kinase
Gopc	Golgi-associated PDZ and coiled-coil motif-containing protein
GPCR	G protein-coupled receptor
iGlu	ionotropic glutamate
IL2	second intracellular loop
INM	inner nuclear membrane
IP_3_	inositol-1,4,5-triphosphate
JNK	c-Jun *N*-terminal kinase
LTD	long-term depression
MAPK	mitogen-activated protein kinase
mGlu	metabotropic glutamate
NAc	nucleus accumbens
NMDA	*N*-methyl-d-aspartate
nRTK	non-receptor tyrosine kinase
PDZ	[PSD95/*Drosophila* disc large tumor suppressor (Dlg1)/zonula occludens-1 (zo-1)]
PKC	protein kinase C
PrP^C^	cellular prion protein
PrP^SC^	scrapie prion protein
PSD	postsynaptic density
SAPK	stress-activated protein kinase
SFK	Src family kinase
SNAP23	synaptosome-associated protein 23
S-SCAM	synaptic scaffolding molecule
SNARE	soluble N-ethylmaleimide-sensitive-factor attachment protein receptor
SNX	sorting nexin
